# Rewiring cell death, metabolism and immunity in precision medicine: insights from the CDDisease 12th Meeting

**DOI:** 10.1038/s41419-026-09017-0

**Published:** 2026-07-21

**Authors:** Peishan Li, Mauro Piacentini, Yufang Shi, Gerry Melino

**Affiliations:** 1 The Fourth Affiliated Hospital of Soochow University, Suzhou Medical College of Soochow University, Biomedical Basic Research Center (BBRC) of Jiangsu Province, Soochow University, Suzhou, Jiangsu China; 2 Department of Experimental Medicine and Biochemical Sciences, University of Rome “Tor Vergata”, Rome, Italy; 3 National Institute for Infectious Diseases IRCCS “Lazzaro Spallanzani”, Rome, Italy

**Keywords:** Cell death, Cancer, Immunology

Following the long tradition of Cell Death & Disease, interrupted only the the COVID-19 events, the CDDisease 12th Meeting: Precision Medicine Symposium, held on April 23–24, 2026 in Suzhou, China, was organized within the framework of the Italy–China bilateral initiative in precision medicine, promoted by the Heal Italia Foundation in collaboration with Cell Death & Disease (Springer Nature), and jointly organized by The Fourth Affiliated Hospital of Soochow University. The meeting, with two magistral keynote lectures by Junying Yuan (Shanghai Institute of Organic Chemistry, China) and John Silke (Walter and Eliza Hall Institute, Australia), brought together leading scientists and clinicians from diverse disciplines, providing a platform for interdisciplinary exchange and reflecting a growing global effort to advance precision medicine through international collaboration.

Precision medicine is undergoing a transition from target-centric approaches to systems-level strategies that integrate genomic, clinical, environmental, and digital information. Beyond its technological foundations, this paradigm reflects a conceptual shift toward understanding disease as the outcome of interconnected biological networks. In this context, the meeting highlighted a unifying principle: cell death pathways function as central regulatory hubs that integrate metabolic and immune signaling to shape disease progression and therapeutic responses.

## Cell death signaling as an integrative regulatory platform

The central role of regulated cell death in human disease was a major focus. In the keynote lecture, Junying Yuan highlighted receptor-interacting protein kinase 1 (RIPK1) as a key regulator balancing pro-survival and pro-death signaling. Beyond its role in regulating TNF-induced cell death, including apoptosis and necroptosis, RIPK1 integrates inflammatory signaling pathways and has been implicated in the pathogenesis of neurodegenerative and inflammatory diseases. The development of selective RIPK1 inhibitors underscores the translational potential of targeting this pathway.

Sudan He (Suzhou Institute of Systems Medicine, China) further refined the molecular framework of RIPK1 activation by identifying an E3 ligase required for RIPK1 ubiquitination in TNF-induced cell death, thereby promoting the formation of pro-death signaling complexes. These findings reveal additional regulatory layers governing the transition between survival and cell death signaling.

At the level of mitochondrial regulation, Quan Chen (Nankai University, China) described the PGAM5–FUNDC1 axis as a molecular switch coordinating mitophagy and apoptosis. By modulating FUNDC1 and Bcl-xL, this pathway links mitochondrial quality control to cellular metabolism and cell fate determination. Complementing these findings, Francis Ka-Ming Chan (Zhejiang University, China) highlighted the dual roles of RIPK3 in both cell death and cytokine signaling in host defense, emphasizing its contribution to tissue homeostasis.

For the second keynote lecture, John Silke reviewed the development of SMAC mimetics targeting inhibitors of apoptosis proteins, emphasizing both their clinical promise and current limitations.

Together, these findings challenge the traditional view of cell death as a terminal event and instead position it as an active regulatory interface linking inflammation, metabolism, and tissue homeostasis.

## Convergent mechanisms in cancer progression and therapy resistance

Cancer progression and therapy resistance emerged as outcomes of multilayered regulatory networks integrating transcriptional, metabolic, and signaling pathways. Qiang Sun (Academy of Military Medical Sciences, China) identified a non-canonical ROS-buffering mechanism in hepatocellular carcinoma (HCC), whereby HIF1α–KLF4 signaling induces ectopic hemoglobin expression to scavenge reactive oxygen species and promote resistance to targeted therapies such as sorafenib.

At the transcriptional level, Gerry Melino (University of Rome “Tor Vergata”, Italy) discussed the role of p63 and its interacting partners in regulating epithelial cell fate and tumor progression, while Giorgio Stassi (University of Palermo, Italy) demonstrated that tumor-associated macrophages drive epigenetic silencing of p53 in colorectal cancer stem cells via a C1Q–IL-6–CEBPD–SETD8 axis. Disruption of this signaling loop restores p53 activity and suppresses tumor growth, highlighting the importance of tumor–immune crosstalk.

Mauro Piacentini (University of Rome “Tor Vergata”, Italy) showed that inhibition of transglutaminase 2 suppresses HCC development by impairing serine biosynthesis and enhancing cell death, with pharmacological inhibition recapitulating genetic ablation. Zhi-Xiong Jim Xiao (Sichuan University, China) further demonstrated that targeting the E3 ligase FBXL2 enables reprogramming of EGFR/HER2 signaling, thereby overcoming resistance in lung cancer and triple-negative breast cancer.

Massimo Dominici (University of Modena and Reggio Emilia, Italy) extended this therapeutic perspective by discussing cell and gene therapy approaches designed to induce tumor cell death in refractory cancers. His presentation highlighted engineered mesenchymal stromal cells and CAR-modified immune cells as complementary platforms to enhance targeted cytotoxicity and potentially reshape the tumor microenvironment.

Collectively, these studies indicate that tumor progression and therapeutic resistance arise from the convergence of signaling, metabolic, and epigenetic networks, while also highlighting emerging therapeutic strategies that induce tumor cell death and reshape the tumor microenvironment.

## Immune regulation across tissue, metabolic, and therapeutic contexts

A major conceptual advance emerging from the meeting is the recognition that immune regulation is shaped not only by classical cytokine and checkpoint pathways, but also by metabolic and tissue-contextual cues.

Chunyi Li (Changchun Sci-Tech University, China) introduced deer antler development as a distinctive model of postnatal organogenesis, showing that androgen-primed antler stem cells recruit macrophages through immunochemotactic signals such as CCL2 to initiate antler formation. These findings highlight the importance of stem cell–immune cell interactions in tissue regeneration.

In cancer immunology, Ling Lu (Xuzhou Medical University, China) identified alanyl-tRNA synthetase 1 (AARS1) as a key regulator linking tumor metabolism to immune suppression. Through protein lactylation-dependent stabilization of ATF6, this pathway promotes kynurenine production and regulatory T cell differentiation, establishing a positive feedback loop that reinforces tumor progression and resistance to PD-1 blockade. Disruption of this circuit enhances immunotherapy responsiveness.

Yu Chen (Fujian Medical University, China) addressed immune-related adverse events associated with immune checkpoint inhibitors, emphasizing their multi-organ involvement, clinical heterogeneity, and current therapeutic limitations. His group has developed organ-specific murine models and multi-omics approaches to support biomarker-driven strategies that balance antitumor efficacy with toxicity management.

In parallel, Ying Wang (Shanghai Institute of Nutrition and Health, China) demonstrated that lipid metabolism regulates stem cell fate and immune development, with SCD1-dependent lipid metabolism controlling stem cell differentiation and influencing thymic epithelial cell regulation of thymocyte differentiation and peripheral regulatory T cell generation. Wei Jia (University of Hong Kong, China) extended these findings to systemic metabolism, showing that bile acid species regulate glucose metabolism, immune development, and microbiota interactions through gut–liver axis pathways.

At the molecular level, Yue Eugene Qin (Zhejiang Provincial People’s Hospital, China) highlighted lysine post-translational modifications as central integrators of metabolic and signaling pathways. Together, these studies support the view that metabolism is a fundamental determinant of immune regulation, tissue homeostasis, and therapeutic response.

## Emerging biomarkers, regulatory layers, and scientific exchange

Advances in biomarker discovery further support precision medicine strategies. Sabrina Giglio (Vita-Salute San Raffaele University, Italy) highlighted the clinical utility of cell-free DNA as a minimally invasive biomarker across oncology and systemic diseases through multi-omic profiling approaches. Andrea Perra (University of Cagliari, Italy) demonstrated the context-dependent role of HLA-G in liver diseases, underscoring its potential as a prognostic biomarker and therapeutic target.

In addition to scientific presentations, the meeting included an online session with the Cell Death & Disease editorial office. Lawal Abiola (Springer Nature, London, UK) provided an overview of the journal’s editorial scope and its emphasis on translational cell death research across cancer, immunity, neuroscience, and metabolism. This session was followed by an interactive discussion addressing publication standards and emerging research priorities, highlighting the importance of aligning mechanistic discoveries with translational impact.

## Conclusion

The CDDisease 12th Meeting highlights a fundamental transition in precision medicine. Rather than focusing on individual molecular targets, current research increasingly emphasizes the integration of cell death pathways, metabolic networks, and immune regulation as interconnected systems that define disease outcomes.

Within this framework, cell death regulators can act as signaling hubs, metabolic programs shape immune landscapes, and microenvironmental interactions influence therapeutic responses. These advances suggest that effective precision medicine will depend on targeting dynamic biological networks rather than isolated molecular pathways. However, a key challenge remains in understanding how these interconnected systems operate across different disease contexts and patient populations. Addressing this challenge will be essential for translating systems-level insights into clinically actionable strategies.

